# The Electro Chemical Theory

**Published:** 1880-08

**Authors:** S. B. Palmer


### ARTICLE III.

### *The Electro-Chemical Theory.*

BY S. B. PALMER, D. D. S.

If we can judge from discussions upon the electro-chemical theory as reported in the journals, and by the letters of inquiry upon the same subject, there is much need of knowledge of the principles involved, the object in view, and application of these principles in daily practice. The space at our command limits this article to a mere digest of the subject.

We claim no more for the doctrines inculcated than science supports; we expect no concessions from opponents that science does not demand. The writer is responsible for the enunciation that failure in operations is mainly due to incompatibility of filling materials with tooth bone. We deem it best to consider but a single principle in this short article, namely: the cause of incompatibility of fillings, and will take for illustration, gold and gutta percha, which are the two extremes. We venture to say, the latter is most compatible with teeth bone under the following conditions: In cases of leaky fillings, and in teeth so frail as to admit moisture to the filling through the tooth alone.

The theory is based upon the universal law that contact, say of copper and zinc, in a battery gives passage to a galvanic current—increased chemical action at the expense or loss of the zinc. Every tooth filled with metal is a miniature battery subject to the same fundamental law. All the variations, from preservation to destruction of teeth by filling, are modifications or change of conditions. The results are the outcome of circumstances elected by the operator in the adaptation of filling material to tooth bone. Moisture is an element of secondary decay or decay around fillings. This is admitted by all opponents to the theory, yet it is said that "failure in operations is mainly due to defective manipulation." Why do defects produce decay? Simply because gold is incompatible with tooth bone in the presence of moisture. The perfect filling deprives the battery of the fluid element and checks the current as in any other battery. The fact that perfect fillings of this material preserve teeth is proof of our position. Imperfection likewise is proof of incompatibility.

Moisture is an element in all teeth, yet all teeth do not decay; neither do any decay from within. Decay around fillings is the result of moisture decomposed. Decomposition is a process of chemical action. Ordinary decay is a manifestation of such action. Galvanism, which is modified electricity, is always present with chemical action. The galvanic current is a powerful agent of decomposition. Now let us consult nature's text-book, the galvanic battery, for facts. We find that when two metals are plunged into a liquid which is a conductor of electricity, and of a nature to act upon one or both of the metals unequally, they will be found to be in different electrical condition—the one positive and the other negative. There is no current produced till contact is made between the two elements, then the current is established and continues as long as chemical action and contact remain.

There has been no little dispute upon the question as to which precedes the other—chemical action or the current. A little reflection would save time in this direction. Each is a part of a circle. The two elements, when separated, are in a state of static electricity. Contact equalizes the potential and but for chemical action they would be equally electrified. But chemical action, which is induced by the current, maintains the inequality and thus the current continues. We now transfer the workings of this law to the oral cavity and we find it to obey as correctly as that of gravitation in its manifestations upon a poor fitting atmospheric plate.

Decomposed fluid we have already stated in the cause of incompatibility of filling material with tooth bone. Continued decomposition is maintained by circulation. If a filling of any material in general use is perfect at the margins and the tooth is of proper density, there is no chemical action, no current, no decay back of the filling. This action, however, is at *first* set up with amalgam, and usually with tin, in all teeth filled with these materials, but for lack of circulation—more moisture to be decomposed, the effects of the effort remain an insoluable compound, compatible with both filling material and dentine. Lest the remarks respecting amalgam may not be understood it should be known that amalgam contains at least two elements of a battery, the alloy and the mercury. The two are not chemically combined like the melting together of gold and silver. The presence of a fluid like oral secretions furnishes the third element of a battery, and sets up slight action with amalgam, as can be clearly demonstrated.

The dentinal walls of any tooth are sufficiently damp to corrode the surface of amalgam in contact therewith. Thus we look in vain for brightness in amalgam in contact with dentine. This has nothing to do with the durability of the filling or preservation of the teeth. One advantage which might be claimed for the tarnished lining is that it reduces conductivity of the filling, and in case of frail dentine, or in the teeth of children where calification is incomplete, the metalic compound is received into the animal or organic substance of the teeth, thereby raising it to the same potential as the filling which cuts off chemical action, just as would happen in the deposition of copper upon the zinc element of a battery. With gold there would be, under the same conditions, no tarnish to lessen conductivity—nothing to restore the tooth, which is the positive element, to the same potential with the gold. On the other hand, any admission of moisture between the gold and dentine is equivalent to decomposition of the fluid and the tooth, according to conditions to be mentioned hereafter.

With gutta percha, or any other non-conducting material, the moisture accessable to the filling and walls of the cavity remains unchanged so far as a current is concerned. There would be none of the increased sensitiveness common to like exposure with gold; the conditions remain the same as would be apparent in like exposures where there are no fillings. One of the hindrances to the reception of truth in relation to chemical laws consists in the unwarranted statement that the tendency of the electro-chemical theory is to degrade the profession, also to discourage the use of gold. We do not attempt to meet any such inconsiderate charges, but rely upon the principle that truth cannot injure a just cause. The writer advocates the use of gold to the extent of its usefulness.

We do not claim that a practice conducted upon a scientific basis would essentially differ from one based upon practical experience; truth is a unit no matter how obtained; and practical knowledge is gained only by years of observation—years of failure as well as success. I believe that the result of every operation is determined by positive law, the conditions of which are present at the commencement: that each law acts within its own prescribed sphere; that it is the dentist's privilege, with a knowledge of natural laws, to so select material and arrange the conditions that success may be among things possible. Without this knowledge the young operator is left to go on in the belief that "failure in operation is mainly due to defective manipulation," and perform operations perhaps in direct violation of law itself. We need more investigation for principles upon which to establish practice, and less effort to maintain practice regardless of principle.

In the hope to convince the reader that the laws which we have already set forth are as much in the interest of a gold practice as they are in the other extreme, we quote from the writings of Dr. Geo. Watt, of Ohio, who is well known to the profession as an earnest advocate of gold, and as strongly opposed to amalgam. His practical knowledge of chemistry enables him to comprehend the subject of oral electricity, while his truthfulness to science prompts him to record his views regardless of their application to this or that practice. In returning to the subject of decomposition of fluids under fillings, Dr. Watt says:

"Saliva has a strong affinity for oxygen, and absorbs it readily from the atmosphere; that it corrodes many of the metals: that it is an electrolyte; and that acidity is generally a result of its decomposition." Again "When a gold plug leaks, there is apt to be galvanic action. The organic matter of the dentine is a conductor, and in this case represents the corroded or zinc side of the battery. Hydrochloric acid decay occurs in such cases, when the buccal fluids are normal, but when ammonia is present, we may have white decay co-operative with it, owing to the formation of nitric acid by electrolysis of the ammonia."

There can be nothing plainer than this statement—it is only a transfer from the laboratory to the oral cavity. It involves no new principle; it does not interfere with practice new or old, except as the operator may be governed by scientific diagnosis to adapt filling materials to the conditions which warrant success. We grant that long practice may furnish the same knowledge; but it is not professional nor progressive to insist that all knowledge shall be acquired by the experimental method and at the needless expense of the patient.—*Odontographic Journal.*

